# Supplementary material for: Public Knowledge and Attitude towards Vitiligo: A Cross-Sectional Survey in Jordan
Source: Int J Environ Res Public Health. 2023 Jun 19;20(12):6183. doi: 10.3390/ijerph20126183 (PMC10298545; doi:10.3390/ijerph20126183)
Supplement: Supplementary file 1 [file ijerph-20-06183-s001.zip › Supplementary Table S1.pdf]

Supplementary Table S1

Regression results using knowledge score as the criterion

Predictor	*b*	*b* 95% CI [LL, UL]	sr^2	sr^2 95% CI [LL, UL]	Fit
(Intercept)	6.55**	[5.31, 7.79]			
**Sex**					
Male	-0.50*	[-0.93, -0.06]	.01	[-.01, .02]	
**Age**					
31-50yrs	0.55	[-0.06, 1.15]	.00	[-.01, .02]	
>50yrs	0.95*	[0.08, 1.82]	.01	[-.01, .02]	
**Marital Status**					
Single	0.12	[-0.50, 0.74]	.00	[-.00, .00]	
**Income**					
Less than 600	-0.37	[-0.84, 0.09]	.00	[-.01, .01]	
More than 1200	-0.21	[-0.74, 0.32]	.00	[-.00, .01]	
**Education**					
High school or less	-0.62	[-1.31, 0.07]	.00	[-.01, .01]	
Higher education	0.30	[-0.24, 0.83]	.00	[-.00, .01]	
**Paternal Education**					
High school or less	0.04	[-0.45, 0.52]	.00	[-.00, .00]	
Higher education	-0.47	[-1.13, 0.20]	.00	[-.01, .01]	
**Maternal Education**					
High school or less	-0.16	[-0.65, 0.34]	.00	[-.00, .00]	
Higher	-0.36	[-1.25, 0.54]	.00	[-.00, .01]	

education				
**Geography**				
Center	-0.11	[-0.82, 0.60]	.00	[-.00, .00]
North	-0.23	[-0.97, 0.51]	.00	[-.00, .00]
South	-0.43	[-0.95, 0.09]	.00	[-.01, .01]
**Location**				
Urban	-0.18	[-0.76, 0.41]	.00	[-.00, .00]
**Occupational Status**				
Student	-0.02	[-0.70, 0.67]	.00	[-.00, .00]
Unemployed	-0.08	[-0.60, 0.43]	.00	[-.00, .00]
**Health Profession**				
Yes	0.69**	[0.17, 1.21]	.01	[-.00, .03]
**Participant has vitiligo**	1.37	[-0.10, 2.84]	.01	[-.01, .02]
**Participant lives with a patient with vitiligo**	0.57	[-0.30, 1.45]	.00	[-.00, .01]
**Participant heard before of vitiligo**	1.65**	[0.80, 2.51]	.02	[.00, .04]
**Participants' partner has vitiligo**	-0.32	[-1.45, 0.80]	.00	[-.00, .00]
**Source of Knowledge**				
Family and Friends	0.68	[-0.09, 1.45]	.00	[-.01, .01]
Books and Magazines	0.42	[-0.49, 1.32]	.00	[-.00, .01]
Internet and Social Media	0.34	[-0.45, 1.14]	.00	[-.00, .01]
Physician	0.54	[-0.79, 1.88]	.00	[-.00, .01]
TV	0.46	[-0.55, 1.48]	.00	[-.00, .01]
Others	0.33	[-0.57, 1.23]	.00	[-.00, .00]

**Attitude Score**	0.17**	[0.11, 0.23]	.05	[.02, .08]	
					R^2 = .198** 95% CI[.10,.21]

Note. A significant *b*-weight indicates the semi-partial correlation is also significant. *b* represents unstandardized regression weights. sr^2 represents the semi-partial correlation squared. *LL* and *UL* indicate the lower and upper limits of a confidence interval, respectively.
* indicates p < .05. ** indicates p < .01.
