# Supplementary material for: Public Knowledge and Attitude towards Vitiligo: A Cross-Sectional Survey in Jordan
Source: Int J Environ Res Public Health. 2023 Jun 19;20(12):6183. doi: 10.3390/ijerph20126183 (PMC10298545; doi:10.3390/ijerph20126183)
Supplement: Supplementary file 1 [file ijerph-20-06183-s001.zip › Supplementary Table S2.pdf]

Supplementary Table S2

Regression results using attitude score as the criterion

Predictor	*b*	*b* 95% CI [LL, UL]	sr^2	sr^2 95% CI [LL, UL]	Fit
(Intercept)	8.98**	[7.04, 10.92]			
**Sex**					
Male	-0.31	[-0.91, 0.30]	.00	[-.00, .01]	
**Age**					
31-50yrs	-1.40**	[-2.24, -0.57]	.02	[-.00, .03]	
>50yrs	-2.31**	[-3.50, -1.11]	.02	[.00, .04]	
**Marital Status**					
Single	0.39	[-0.47, 1.25]	.00	[-.00, .01]	
**Income**					
Less than 600	-0.43	[-1.07, 0.22]	.00	[-.00, .01]	
More than 1200	0.32	[-0.42, 1.06]	.00	[-.00, .01]	
**Education**					
High school or less	1.10*	[0.14, 2.05]	.01	[-.01, .02]	
Higher education	-0.24	[-0.98, 0.51]	.00	[-.00, .00]	
**Paternal Education**					
High school or less	-0.10	[-0.78, 0.57]	.00	[-.00, .00]	
Higher education	0.51	[-0.41, 1.44]	.00	[-.00, .01]	
**Maternal Education**					
High school or less	-0.02	[-0.71, 0.67]	.00	[-.00, .00]	
Higher education	0.22	[-1.02, 1.47]	.00	[-.00, .00]	
**Geography**					
Center	0.31	[-0.68, 1.29]	.00	[-.00, .00]	
North	-0.97	[-2.00, 0.05]	.00	[-.01, .02]	
South	-0.57	[-1.28, 0.15]	.00	[-.01, .01]	
**Location**					
Urban	0.72	[-0.09, 1.53]	.00	[-.01, .01]	

**Occupational Status**					
Student	0.06	[-0.89, 1.01]	.00	[-.00, .00]	
Unemployed	0.26	[-0.46, 0.98]	.00	[-.00, .00]	
**Health Profession**					
Yes	-0.05	[-0.78, 0.68]	.00	[-.00, .00]	
**Participant has vitiligo**	-0.28	[-2.33, 1.77]	.00	[-.00, .00]	
**Participant lives with a patient with vitiligo**	1.62**	[0.41, 2.83]	.01	[-.00, .02]	
**Participant heard before of vitiligo**	2.07**	[0.88, 3.26]	.02	[-.00, .04]	
**Participants' partner has vitiligo**	-1.05	[-2.61, 0.52]	.00	[-.00, .01]	
**Source of Knowledge**					
Family and Friends	-0.26	[-1.34, 0.81]	.00	[-.00, .00]	
Books and Magazines	0.04	[-1.22, 1.30]	.00	[-.00, .00]	
Internet and Social Media	-0.63	[-1.74, 0.47]	.00	[-.00, .01]	
Physician	0.65	[-1.20, 2.50]	.00	[-.00, .00]	
TV	-1.34	[-2.75, 0.07]	.01	[-.01, .02]	
Others	-0.28	[-1.53, 0.97]	.00	[-.00, .00]	
**Knowledge Score**	0.31**	[0.19, 0.42]	.04	[.01, .07]	
					R^2 = .236**
					95% CI[.13,.25]

Note. A significant *b*-weight indicates the semi-partial correlation is also significant. *b* represents unstandardized regression weights. sr^2 represents the semi-partial correlation squared. *LL* and *UL* indicate the lower and upper limits of a confidence interval, respectively.

* indicates p < .05. ** indicates p < .01.
