# Supplementary material for: Public Knowledge and Attitude towards Vitiligo: A Cross-Sectional Survey in Jordan
Source: Int J Environ Res Public Health. 2023 Jun 19;20(12):6183. doi: 10.3390/ijerph20126183 (PMC10298545; doi:10.3390/ijerph20126183)
Supplement: Supplementary file 1 [file ijerph-20-06183-s001.zip › Supplementary Table S3.pdf]

Supplementary table S3

Participants' responses to questions regarding the knowledge of vitiligo.

Questions		Count	%
**Is vitiligo contagious?**	No*	862	86.72
	Maybe	104	10.46
	Yes	28	2.82
**Is vitiligo a hereditary disease?**	No*	244	24.55
	Maybe	326	32.80
	Yes	424	42.66
**Is vitiligo an autoimmune disease?**	No	311	31.29
	Maybe	307	30.89
	Yes*	376	37.83
**Does vitiligo affect skin?**	No	33	3.32
	Maybe	141	14.19
	Yes*	820	82.49
**Is vitiligo hygiene-related?**	No*	873	87.83
	Maybe	93	9.36

	Yes	28	2.82
**Does vitiligo affect internal organs?**	No*	407	40.95
	Maybe	355	35.71
	Yes	232	23.34
**Is vitiligo caused by certain foods?**	No*	796	80.08
	Maybe	171	17.20
	Yes	27	2.72
**Does vitiligo cause skin cancer?**	No*	586	58.95
	Maybe	337	33.90
	Yes	71	7.14
**Does vitiligo lead to death?**	No*	884	88.93
	Maybe	96	9.66
	Yes	14	1.41
**Is vitiligo caused by contaminated foods?**	No*	836	84.1
	Maybe	140	14.08
	Yes	18	1.81
**Is vitiligo triggered by psychological stress?**	No	262	26.36

	Maybe	361	36.32
	Yes*	371	37.32
**Is vitiligo triggered by magic or witchery?**	No*	829	83.4
	Maybe	145	14.59
	Yes	20	2.01
**Does vitiligo affect the social life of patients?**	No	339	34.10
	Maybe	313	31.49
	Yes*	342	34.41
**Is there a treatment for vitiligo?**	No	293	29.48
	Maybe	425	42.76
	Yes*	276	27.77

*Correct answers
