# Supplementary material for: Public Knowledge and Attitude towards Vitiligo: A Cross-Sectional Survey in Jordan
Source: Int J Environ Res Public Health. 2023 Jun 19;20(12):6183. doi: 10.3390/ijerph20126183 (PMC10298545; doi:10.3390/ijerph20126183)
Supplement: Supplementary file 1 [file ijerph-20-06183-s001.zip › Supplementary Table S4.pdf]

Supplementary Table S4

Participants' responses to questions regarding the attitudes toward vitiligo N (%).

statements	Yes	Maybe	No
**I would eat food prepared by a vitiligo patient**	803 (80.78)	119 (11.97)	72 (7.24)
**I would become a friend with a vitiligo patient**	803 (80.78)	119 (11.97)	72 (8.08)
**I would marry a vitiligo patient**	336 (33.8)	301 (30.28)	357 (35.92)
**I would marry a family member who has vitiligo**	377 (37.93)	288 (28.97)	329 (33.1)
**My intimate relationship would not be affected by my partners' vitiligo diagnosis**	634 (64.50)	210 (21.36)	139 (14.14)
**I would not want to divorce if my partner got diagnosed with vitiligo**	850 (85.51)	93 (9.36)	51 (5.13)
**I would shake hands with a vitiligo patient**	874 (87.93)	72 (7.24)	48 (4.83)
**I would hire a vitiligo patient**	839 (84.41)	116 (11.67)	39 (3.92)
